# Single-Trial Decoding of Bistable Perception Based on Sparse Nonnegative Tensor Decomposition

**DOI:** 10.1155/2008/642387

**Published:** 2008-05-22

**Authors:** Zhisong Wang, Alexander Maier, Nikos K. Logothetis, Hualou Liang

**Affiliations:** ^1^School of Health Information Sciences, University of Texas Health Science Center at Houston, 7000 Fannin, Suite 600, Houston, TX 77030, USA; ^2^Unit on Cognitive Neurophysiology and Imaging, National Institute of Health, Building 49, Room B2J-45, MSC-4400, 49 Convent Dr., Bethesda, MD 20892, USA; ^3^Max Planck Institut für biologische Kybernetik, Spemannstrasse 38, 72076 Tübingen, Germany

## Abstract

The study of the neuronal correlates of the spontaneous alternation in perception elicited by bistable visual stimuli is promising for understanding the mechanism of neural information processing and the neural basis of visual perception and perceptual decision-making. In this paper, we develop a sparse nonnegative tensor factorization-(NTF)-based method to extract features from the local field potential (LFP), collected from the middle temporal (MT) visual cortex in a macaque monkey, for decoding its bistable structure-from-motion (SFM) perception. We apply the feature extraction approach to the multichannel time-frequency representation of the intracortical LFP data. The advantages of the sparse NTF-based feature extraction approach lies in its capability to yield components common across the space, time, and frequency domains yet discriminative across different conditions without prior knowledge of the discriminating frequency bands and temporal windows for a specific subject. We employ the support vector machines (SVMs) classifier based on the features of the NTF components for single-trial decoding the reported perception. Our results suggest that although other bands also have certain discriminability, the gamma band feature carries the most discriminative information for bistable perception, and that imposing the sparseness constraints on the nonnegative tensor factorization improves extraction of this feature.

## 1. Introduction

The question of cortex is of central importance to many issues in cognitive neuroscience. To answer this question, one important experimental paradigm is to dissociate percepts from the visual inputs using bistable stimuli. The study of bistable perception holds great promise for understanding the neural correlates of visual perception [1]. Spiking activity has been extensively studied in brain research to determine the relationship between perceptual reports during ambiguous visual stimulation in the middle temporal area (MT) of macaque monkeys [2, 3]. However, spiking data as collected with standard neurophysiological techniques only provide information about the outputs of a small number of neurons within a given brain area. The local field potential (LFP) has recently attracted increasing attention in the analysis of the neuronal population activity [4, 5]. LFP is thought to largely arise from the dendritic activity of local populations of neurons and is dominated by the excitatory synaptic inputs to a cortical area as well as intra-areal local processing. The investigation of the correlations between perceptual reports and LFP oscillations during physically identical but perceptually ambiguous conditions may shed new lights on the mechanism of neural information processing and the neural basis of visual perception and perceptual decision-making.

One important research direction in the field of neuroscience is to study the rhythmic brain activity during different tasks. For example, it is discovered that the beta and mu bands are associated with event-related desynchronization and the gamma band is associated with event-related synchronization for movement and motor imaginary tasks [6, 7], and that the gamma band is also associated with memory and attention [4, 8]. The brain oscillations for bistable perceptual discrimination, on the other hand, are not easy to distinguish and it remains largely unknown which band is the most discriminative for bistable perception. In line with the recent literature, in this paper, we discover that the gamma oscillation is particularly discriminative for distinguishing different percepts. For neurobiological time series, theunderlying processes are often nonstationary. To reveal the temporal structure of LFP, the LFP spectrum at a certain time and frequency is often analyzed. For example, the short-time Fourier transform (STFT) provides a means of joint time-frequency analysis by applying moving windows to the signal and Fourier transforming the signal within each window [9]. With technological advances, multichannel intracortical recordings become available nowadays and they provide new opportunities to study how populations of neurons interact to produce a certain perceptual outcome. However, different channels of LFP may record not only brain activity correlated with the percept but also background ongoing activity that is not percept-correlated. It is of interest to decompose the multichannel time-varying LFP spectrum into multiple components with distinct modalitiesin the space, time, and frequency domains to identify among them the components common across different domains and at the same time discriminative across different conditions.

The conventional two-way decomposition approaches include principal component analysis (PCA), independent component analysis (ICA), and linear discriminant analysis (LDA), which extract features from two-way data (matrices) by decomposing them into different factors (modalities) based on orthogonality, independence, and discriminability, respectively. However, PCA, ICA, or LDA all represent data in a holistic way with their factors both additively and subtractively combined. For two-way decomposition of nonnegative data matrices, it is intuitive to allow only nonnegative factors to achieve an easily interpretable parts-based representation of data. Such an approach is called nonnegative matrix factorization (NMF) [10, 11]. In practical applications, multiway data (tensors) with three or more modalities often exist. If two-way decomposition approaches are to be used under these circumstances, tensors have to be first converted into matrices by unfolding several modalities. However, such unfolding may lose some information specific to the unfolded modalities and make it less easy to interpret the decomposed components. Therefore, to obtain a more natural representation of the original data structure, it is recommended to use tensor decomposition approaches to factorize multiway data. PARAFAC and TUCKER models are typical models for tensor factorization [12–14]. Their difference lies in that the TUCKER model permits the interactions within each modality while the PARAFAC model does not. The PARAFAC model is often used due to two advantages it possesses. First, it is the simplest and most parsimonious multiway model and hence its parameter estimation is easier than all the other multiway models. Second, it can achieve unique tensor decomposition up to trivial permutation, sign changes, and scaling as long as several weak conditions are satisfied [15, 16]. In neuroscientific applications, the PARAFAC model was used to analyze the three-way space-time-frequency representation of the EEG data [17, 18]. However, the original PARAFAC model does not assume nonnegative constraints on its factors. As a result, in some cases the estimated PARAFAC model for the nonnegative tensor data may be difficult to interpret. The nonnegative tensor factorization (NTF), as its name implies, enforces the nonnegative constraint on each modality and is more appropriate for decomposing nonnegative tensor data. In fact, NTF has been widely used in diverse fields ranging from chemometrics, image analysis, signal processing, to neuroscience [19–25]. For example, in [23], the PARAFAC model with nonnegative constraints was used to decompose the multiway intertrial phase coherence (ITPC) defined in [26], which is the average of the normalized space-time-frequency representation of data across trials. For single-trial decoding, however, features have to be extracted from each single trial and hence ITPC cannot be used. It is worthy to mention that there is a possible expense associated with the imposition of the nonnegative constraints on the the PARAFAC model, namely the loss of uniqueness in the decomposition [27]. Nevertheless, sparseness constraints can be enforced to improve the uniqueness of the nonnegatively constrained PARAFAC decomposition and remarkably, sparseness constraints can enhance the parts-based representation of the data [28, 29].

In this paper, we develop a sparse NTF-based method to extract features from the LFP responses for decoding the bistable structure-from-motion (SFM) perception. We apply the feature extraction approach to the multichannel time-frequency representation of intracortical LFP data collected from the MT visual area in a macaque monkey performing a SFM task, aiming to identify components common across the space, time, and frequency domains and at the same time discriminative across different conditions. To determine the best LFP band for bistable perceptual discrimination, we first cluster each NTF component using the *K*-means clustering algorithm based on its frequency modality that measures the spectral characteristics of the component, and then employ a support vector machines (SVMs) classifier to decode the monkey's perception on a single-trial basis to determine the discriminability of each cluster. In doing so, we have discovered that although other bands also have certain discriminability, the gamma band feature carries the most discriminative information for bistable perception, and that imposing the sparseness constraints on the nonnegative tensor factorization improves extraction of this feature. The rest of the paper is organized as follows. In Section 2, we first present the experimental paradigm and then introduce the sparse NTF approach, the *K*-means clustering algorithm, and the SVM classifier. In Section 3, we explore the application of the NTF-based approach for decoding the bistable SFM perception. Finally, Section 4 contains the conclusions.

## 2. Materials and Methods

### 2.1. Subjects and Neurophysiological Recordings

Electrophysiological recordings were performed in a healthy adult male rhesus monkey. After behavioral training was complete, occipital recording chambers were implanted and a craniotomy was made. Intracortical recordings were conducted with a multielectrode array while the monkey was viewing structure-from-motion (SFM) stimuli, which consisted of an orthographic projection of a transparent sphere that was covered with randomly distributed dots on its entire surface. Stimuli rotated for the entire period of presentation, giving the appearance of three-dimensional structure. The monkey was well trained and required to indicate the choice of rotation direction (clockwise or counterclockwise) by pushing one of two levers. Correct responses for disparity-defined stimuli were acknowledged with application of a fluid reward. In the case of fully ambiguous (bistable) stimuli, where the stimuli can be perceived in one of two possible ways and no correct response can be externally defined, the monkey was rewarded by chance. Only the trials of data corresponding to bistable stimuli are analyzed in the paper. The recording site was the middle temporal area (MT) of the monkey's visual cortex, which is commonly associated with visual motion processing. LFP was obtained by filtering the collected data between 1 to 100 Hz.

### 2.2. Sparse Nonnegative Tensor Factorization

In [11], two algorithms with multiplicative factor updates were proposed to solve the NMF problem. One algorithm is based on minimization of the squared error, while the other is based on minimization of the generalized Kullback-Leibler (KL) divergence. These algorithms were extended to the NTF problem using the PARAFAC model in [21]. Sparseness constraints originally proposed for NMF [28, 29] can also be incorporated in NTF to enhance the uniqueness of the nonnegatively constrained PARAFAC decomposition and improve the parts-based representation of the data. In the paper, we focus on a sparse NTF algorithm based on the nonnegatively and sparsely constrained PARAFAC model and minimization of the generalized KL divergence. The sparseness constrains imposed are similar to those of [28].

Let *𝒳* ∈ *ℝ* ^*I*_1_×*I*_2_×⋯×*I*_*N*_^ denote an *N*-way tensor with *N* indices (*i*_1_ *i*_2_⋯*i*_*N*_). Let *𝒳*_*i*_1_*i*_2_⋯*i*_*N*__ represent an element with 1 ≤ *i*_*n*_ ≤ *I*_*n*_. Assume that the PARAFAC model decomposes the tensor *𝒳* into *K* components, each of which is the outer product of vectors that span different modalities,

(1) $${𝒳}_{i_{1}i_{2}\cdots i_{N}}\approx \overset{K}{\underset{k=1}{\sum }}A_{i_{1}k}^{\left(1\right)}A_{i_{2}k}^{\left(2\right)}\cdots A_{i_{N}k}^{\left(N\right)},$$

where **A** ^(*n*)^ ∈ *ℝ* ^*I*_*n*_×*K*^ is the matrix corresponding to the *n*th modality.

A tensor can be converted into a matrix. Let the matrix **X**_(*n*)_ ∈ *ℛ* ^*I*_*n*_×*I*_1_⋯*I*_*n*−1_*I*_*n*+1_⋯*I*_*N*_^ denote the mode-*n* matricization of *𝒳*. Then it follows

(2) $$X_{\left(n\right)}\approx A^{\left(n\right)}Z_{\left(n\right)}$$

with

(3) $$Z_{\left(n\right)}={\left(A^{\left(N\right)}\left|⊗\right|\cdots |⊗|\;A^{\left(n+1\right)}|⊗|\;A^{\left(n-1\right)}|⊗|\cdots |⊗|\;A^{\left(1\right)}\right)}^{T},$$

where |⊗| denotes the Khatri-Rao product (column-wise Kronecker product) and (⋅)^*T*^ means transpose.

The cost function for the sparse NTF approach based on minimization of the generalized KL divergence can be written as

(4) $$\begin{array}{c} \underset{ij}{\sum }({\left(X_{\left(n\right)}\right)}_{ij}\log\frac{{\left(X_{\left(n\right)}\right)}_{ij}}{{\left(A^{\left(n\right)}Z_{\left(n\right)}\right)}_{ij}}-{\left(X_{\left(n\right)}\right)}_{ij}\\ \;+{\left(A^{\left(n\right)}Z_{\left(n\right)}\right)}_{ij})+\lambda \underset{ij}{\sum }{\left(A^{\left(n\right)}\right)}_{ij}, \end{array}$$

where *λ* is the regularization parameter for the sparse constraints. Note that if *λ* = 0, this corresponds to the nonsparse NTF approach. The factor update for the sparse NTF approach is the same as that in [11] except an extra regularization term;

(5) $$\begin{array}{cc} A^{\left(n\right)} & =A^{\left(n\right)}⊙\left(X_{\left(n\right)}⊘\left(A^{\left(n\right)}Z_{\left(n\right)}\right)\right)\\ & \;⊙Z_{\left(n\right)}^{T}⊘\left(A^{\left(n\right)}Z_{\left(n\right)}Z_{\left(n\right)}^{T}+\lambda E\right), \end{array}$$

where **E** is a matrix of ones, ⊙ and ⊘ denote element-wise multiplication and division, respectively. We can first randomly initialize **A** ^(*n*)^,*n* = 1, 2,…, *N* and then alternately update them in an iterative way until convergence. In [11], it was proved that such iterative multiplicative update can be regarded as a special kind of gradient descent update using the optimal step size at each iteration, which is guaranteed to reach a locally optimal factorization.

### 2.3. *K*-means Clustering

The *K*-means clustering algorithm partitions a data set into *K* clusters with each cluster represented by its mean such that the data within each cluster are similar but the data across distinct clusters are different [30]. Initially, the *K*-means clustering algorithm generates *K* random points as cluster means. Then it iterates two steps namely the assignment step and update step until convergence. In the assignment step, each data point is assigned to the cluster so that the distance from the data point to the mean of the cluster is smaller than that from the data point to the means of other clusters. In the update step, the means of all clusters are recomputed and updated based on the data points assigned to them. The convergence criterion can be that the cluster assignment does not change. The *K*-means clustering algorithm is simple and fast but the clustering results depend on the initial random assignments. To overcome this problem, we can take the best clustering from multiple random starts.

We use the silhouette value to determine the number of clusters [31]. The silhouette value measures how similar a data point is to points in its own cluster compared to points in other clusters and is defined as follows:

(6) $$s(i)=(\underset{l}{\min}\;b(i,l)-a(i))/\max(a(i),\underset{l}{\min}\;b(i,l)),$$

where *a*(*i*) is the average distance from the *i*th data point to the other points in its cluster, and *b*(*i*, *l*) is the average distance from the *i*th point to points in another cluster *l*. The silhouette value ranges from −1 to +1 with 1 meaning that data are separable and correctly clustered, 0 denoting poor clustering, and −1 meaning that the data are wrongly clustered.

### 2.4. Support Vector Machines Classifier

Support vector machines (SVMs) is a popular classifier that minimizes the empirical classification error and at the same time maximizes the margin by determining a linear separating hyperplane to distinguish different classes of data [32, 33]. SVM is robust to outliers and has good generalization ability. Consequently, it has been used in a wide range of applications.

Assume that **x**_*k*_, *k* = 1,…, *K* are the *K* training feature vectors for decoding and the class labels are *y*_*k*_ ∈ {−1,+1}, then SVM solves the following optimization problem:

(7) $$\begin{array}{c} \min∥\;w{\;∥}^{2}+C\overset{K}{\underset{k=1}{\sum }}\xi_{k}\;\text{subject to}\\ y_{k}(w^{\prime }x_{k}+b)\geq 1-\xi_{k},\\ \xi_{k}\geq 0, \end{array}$$

where **w** is the weight vector, *C* > 0 is the penalty parameter of the error term chosen by cross-validation, *ξ*_*k*_ is the slack variable, and *b* is the bias term. It turns out that the margin of the two classes is inversely proportionally to ||**w**||^2^. Therefore, the first term in the objective function of SVM is used to maximize the margin. The second term in the objective function is the regularization term that allows for training errors for the inseparable case.

The Lagrange multiplier method can be used to find the optimal solution for *w* and *b* in the above optimization problem. Assume that **t** is the testing feature vector. Then testing is done simply by determining on which side of the separating hyperplane **t** lies, that is, if **w**′**t** + *b* ≥ 0, the label of **t** is classified as +1, otherwise, the label is classified as −1. SVM can also be used as a kernel-based method when the feature vectors are mapped into a higher dimensional space [32].

## 3. Experimental Results

In this section, we provide experimental examples to demonstrate the performance of the proposed feature extraction approach for predicting perceptual decisions from the neuronal data. Simultaneously, collected 4-channel LFP data were used for demonstration. Gabor transform (STFT with a Gaussian window) is used to obtain the time-frequency representation of the data. The number of trials is 96. The time window used is from stimulus onset to 1 second after that. We find that the performance does not change much if a different time window, for example, from stimulus onset to 800 milliseconds after that, is used. We use both nonsparse and sparse NTF approaches based on minimizing the generalized KL divergence and choose the number of NTF components to be 20 with random initialization for all modalities. The regularization parameter *λ* for the sparse NTF approach is chosen to be 0.5 and the sparseness constraint is applied to each modality. We apply the nonsparse and sparse NTF approaches to the nonnegative four-way data (channel by frequency by time by trial) and use the modality corresponding to the trials as the features. We use *K*-means clustering to cluster the features with 50 random starts to find the best clustering and adopt the correlation between the spectral modalities of the NTF components as the distance metric. The NTF and clustering are performed on all the data since they are unsupervised and does not require any label information. On the other hand, if a feature extraction method requires label information, it should be done on the training data only. We employ the linear SVM classifier from the LIBSVM package [34] and use decoding accuracy as the performance measure, calculated via leave-one-out cross-validation (LOOCV). In particular, for a data set with *N* trials, we choose *N* − 1 trials for training and use the remaining 1 trial for testing. This is repeated for *N* times with each trial serving for testing once. The decoding accuracy is obtained as the ratio of the number of correctly decoded trials to *N*. It is also possible to split the data into three disjoint sets: one for parameter estimation, one for model selection, and one for testing the end result. We have considered this option in the past but we decided to use the LOOCV procedure due to the limited number of trials available.

**Figure 1:**
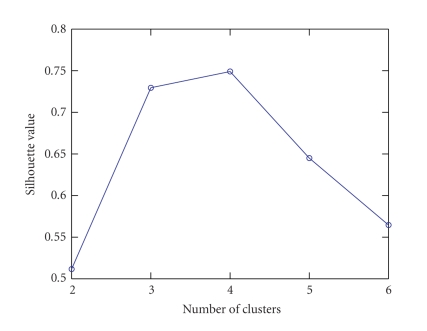
The silhouette value obtained by clustering the nonsparse NTF components using the *K*-means algorithm as a function of the number of clusters.

**Figure 2:**
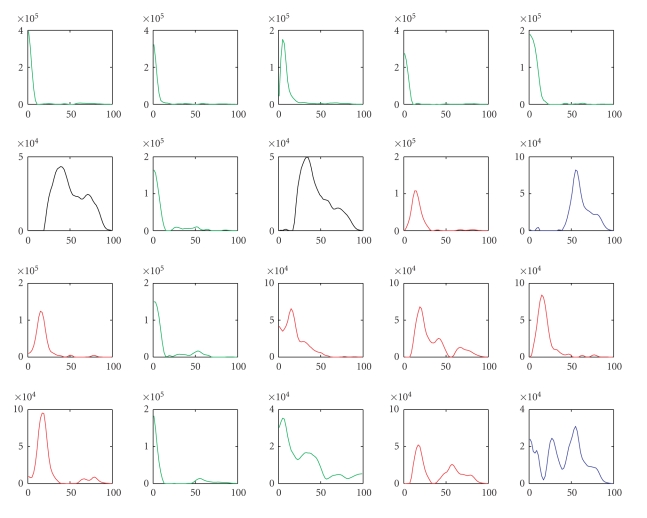
Comparison of the frequency modalities of the 20 nonsparse NTF components clustered by the *K*-means algorithm. The color of each curve denotes to which cluster the component belongs. Blue, green, red, and black correspond to clusters 1–4, respectively.

**Figure 3:**
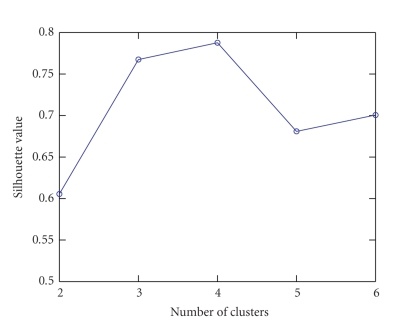
The silhouette value obtained by clustering the sparse NTF components using the *K*-means algorithm as a function of the number of clusters.

**Figure 4:**
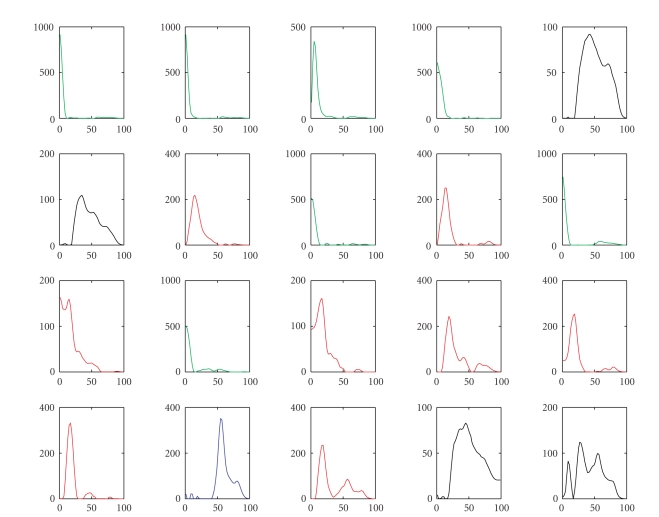
Comparison of the frequency modalities of the 20 sparse NTF components clustered by the *K*-means algorithm. The color of each curve denotes to which cluster the component belongs. Blue, green, red, and black correspond to clusters 1–4, respectively.

Figure 1 shows the silhouette value obtained by clustering the nonsparse NTF components using the *K*-means algorithm as a function of the number of clusters. Note that the silhouette value increases with the number of clusters until the number of clusters is equal to four. Hence we choose the number of clusters to be four. Figure 2 shows the frequency modalities of the 20 nonsparse NTF components clustered by the *K*-means algorithm. The color of each curve denotes to which cluster the component belongs. Blue, green, red, and black correspond to clusters 1–4, respectively. Figures 3 and 4 are the same as Figures 1 and 2, respectively, except that sparse NTF components are used. For comparison, we use the same range for *y* axis in Figure 3 as in Figure 1. Note that the silhouette values of Figure 3 follow a similar trend to that in Figure 1. Hence the number of clusters for the sparse NTF components is also chosen to be four. In addition, it is clear that for a given number of clusters, the silhouette value of Figure 3 is always larger than that of Figure 1. This indicates that the clustering of the sparse NTF components is better than the clustering of the nonsparse NTF components, though the main purpose of these two figures is to show that with NTF method, either sparse or nonsparse, the number of clusters converges to 4. It can be seen from Figures 2 and 4 that both the sparse and nonsparse NTF components are well clustered by the *K*-means algorithm and that different clusters may have different number of components. Furthermore, in both cases, the clusters generally fall into distinct spectral bands: the first cluster mainly in the high gamma band (50–60 Hz), the second cluster in the delta band (1–4 Hz), the third cluster in the alpha band (10–20 Hz), and the fourth cluster mainly in the low gamma band (30–40 Hz).

**Figure 5:**
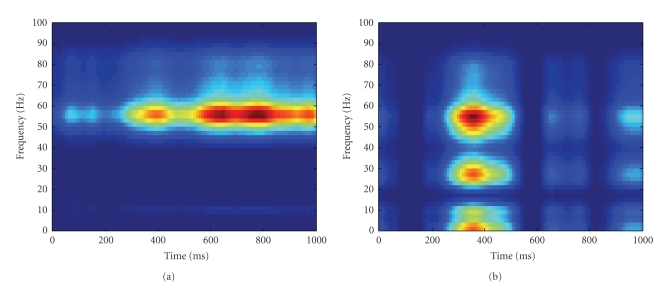
The time-frequency plot for (a) the first nonsparse NTF component and (b) the second nonsparse NTF component of cluster 1. Red and blue represent strong and weak activity, respectively. Note that the first component has localized time-frequency representation in the high gamma band, while the second component contains strong activity in both high gamma band and other bands. In addition, these two components occupy different time windows.

**Figure 6:**
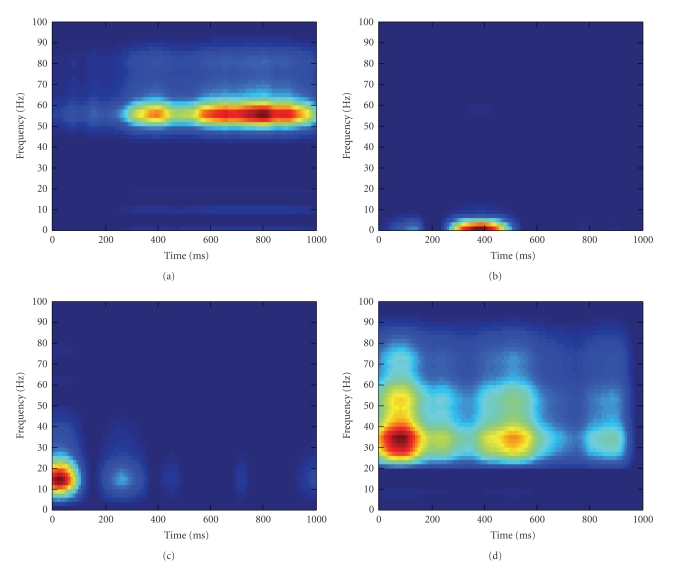
The representative time-frequency plot for (a) cluster 1, (b) cluster 2, (c) cluster 3, and (d) cluster 4, respectively, of the sparse NTF components. Red and blue represent strong and weak activity, respectively. Note that the first cluster for the sparse NTF components contains only one component in the high gamma band (50–60 Hz) with well-localized time-frequency representation, and that clusters 2–4 have concentrated time-frequency distributions in the delta band (1–4 Hz), alpha band (10–20 Hz), and low gamma band (30–40 Hz), respectively.

To have a closer look at the NTF components, we construct the time-frequency representation for each component based on the outer product of its frequency modality and time modality. Figures 5(a) and 5(b) show the time-frequency plot for the two nonsparse NTF components of cluster 1. Red and blue in the figures represent strong and weak activity, respectively. Note that the first nonsparse NTF component has localized time-frequency representation in the high gamma band, while the second component contains strong activity in both the high gamma band and other bands. In addition, these two components cover different time windows with the first component in both an early window and a late window and the second component mainly in an early window. Figures 6(a) and 6(b) show the representative time-frequency plot for (a) cluster 1, (b) cluster 2, (c) cluster 3, and (d) cluster 4, respectively, of the sparse NTF components. Red and blue represent strong and weak activity, respectively. Note the similarity between Figures 6(a) and 5(a). However, unlike the first cluster for the nonsparse NTF components, the first cluster for the sparse NTF components has only one component with well-localized time-frequency representation in the high gamma band (50–60 Hz). From Figures 6(b) to 6(d), we can observe concentrated time-frequency distributions for the second cluster in the delta band (1–4 Hz), the third cluster in the alpha band (10–20 Hz), and the fourth cluster mainly in the low gamma band (30–40 Hz).

We next compare the SVM decoding accuracy based on different features of the nonsparse and sparse NTF components in Tables 1 and 2, respectively. In particular, we compare the decoding accuracy based on the combination of all features from each of clusters 1–4 (denoted as c1 (combined)–c4 (combined), resp.) and the single best feature from each of clusters 1–4 (denoted as c1 (best)–c4 (best), resp.). It is clear that cluster 1 significantly outperforms clusters 2–4 in terms of decoding accuracy. Therefore, the high gamma band feature is more discriminative than the features in the other bands for bistable perception. Note that the combination of all features within one cluster sometimes results in lower decoding accuracy than the single best feature from that cluster. This is probably due to the redundancy of features within the same cluster. Comparing Tables 1 and 2, we can see that the high gamma band feature of the sparse NTF approach is better than that of the nonsparse NTF approach. The former has the best decoding accuracy of 0.76 (corresponding to the sparse NTF component in Figure 6(a)), while the latter has the best decoding accuracy of 0.72 (corresponding to the first nonsparse NTF component in Figure 5(a)). The decoding accuracy for the second nonsparse NTF component of cluster 1 (corresponding to Figure 5(b)) is only 0.61. The decoding performances reveal that although Figures 6(a) and 5(a) appear quite similar, the high gamma band features extracted by the sparse and nonsparse NTF approaches are different. This is due to the fact that the sparseness constraints enhance the parts-based representation of the data and contribute to a better extraction of the high gamma band feature, leading to the improvement of decoding accuracy. We have performed the statistical tests to compare the performances of the sparse and nonsparse NTF methods. Although in most cases, there is no significant difference between them, the sparse NTF significantly outperforms the nonsparse NTF in the case of the combination of features for the high gamma frequency band. Furthermore, the results of both the sparse NTF and nonsparse NTF show significant difference between the high gamma frequency band and the other bands. As a benchmark, we have also calculated the SVM decoding accuracy based on the power of bandpass filtered LFP in the frequency bands of commonly used ranges; delta band (1–4 Hz), theta band (5–8 Hz), alpha band (9–14 Hz), beta band (15–30 Hz), and gamma band (30–80 Hz), and found that the maximum decoding accuracy of all is 0.61. Taken together, our results suggest that NTF is useful for LFP feature extraction and that although other bands also have certain discriminability, the gamma band feature carries the most discriminative information for bistable perception, and that imposing the sparseness constraints on the nonnegative tensor factorization improves extraction of this feature.

## 4. Conclusions

In this paper, we have developed a sparse nonnegative tensor factorization-(NTF)-based method to extract features from the local field potential (LFP) in the middle temporal area (MT) of a macaque monkey performing a bistable structure-from-motion (SFM) task. We have applied the feature extraction approach to the multichannel time-frequency representation of the LFP data to identify components common across the space, time, and frequency domains and at the same time discriminative across different conditions. To determine the most discriminative band of LFP for bistable perception, we have clustered the NTF components using the *K*-means clustering algorithm and employed a support vector machines (SVMs) classifier to determine the discriminability of each cluster based on single-trial decoding of the monkey's perception. Using these techniques, we have demonstrated that although other bands also have certain discriminability, the gamma band feature carries the most discriminative information for bistable perception, and that imposing the sparseness constraints on the nonnegative tensor factorization improves extraction of this feature.

## Figures and Tables

**Table 1:** Comparison of the decoding accuracy based on the combination of all features from each of cluster 1–4 (denoted as c1 (combined)–c4 (combined), resp.), and the single best feature from each of cluster 1–4 (denoted as c1 (best)–c4 (best), resp.). Clusters 1–4 correspond to high gamma band (50–60 Hz), delta band (1–4 Hz), alpha band (10–20 Hz), and low gamma band (30–40 Hz), respectively. The nonsparse NTF approach based on minimization of the generalized KL divergence is used.

Feature	c1 (combined)	c2 (combined)	c3 (combined)	c4 (combined)
Decoding accuracy	**0.70**	0.61	0.63	0.63
Feature	c1 (best)	c2 (best)	c3 (best)	c4 (best)
Decoding accuracy	**0.72**	0.61	0.61	0.61

**Table 2:** Comparison of the decoding accuracy based on the combination of all features from each of cluster 1–4 (denoted as c1 (combined)–c4 (combined), resp.), and the single best feature from each of cluster 1–4 (denoted as c1 (best)–c4 (best), resp.). Clusters 1–4 correspond to high gamma band (50–60 Hz), delta band (1–4 Hz), alpha band (10–20 Hz), and low gamma band (30–40 Hz), respectively. The sparse NTF approach based on minimization of the generalized KL divergence is used.

Feature	c1 (combined)	c2 (combined)	c3 (combined)	c4 (combined)
Decoding accuracy	**0.76**	0.61	0.53	0.58
Feature	c1 (best)	c2 (best)	c3 (best)	c4 (best)
Decoding accuracy	**0.76**	0.61	0.61	0.61
